# Effects of Isosorbide Incorporation into Flexible Polyurethane Foams: Reversible Urethane Linkages and Antioxidant Activity

**DOI:** 10.3390/molecules24071347

**Published:** 2019-04-05

**Authors:** Se-Ra Shin, Jing-Yu Liang, Hoon Ryu, Gwang-Seok Song, Dai-Soo Lee

**Affiliations:** 1 Division of Semiconductor and Chemical Engineering, Chonbuk National University, 567 Baekjedaero, Deokjin-gu, Jeonju 54896, Korea; srshin89@jbnu.ac.kr (S.-R.S.); liangjy@naver.com (J.-Y.L.); 2 Industrial Biotechnology Program, Chemical R&D Center, Samyang Corporation, Daedeok-daero 730, Yuseong-gu, Daejeon 34055, Korea; hoon.ryu@samyang.com (H.R.); gwangseok.song@samyang.com (G.-S.S.)

**Keywords:** isosorbide, reversible urethane linkages, cell opening, antioxidant activity, radical scavenger, flexible polyurethane foam

## Abstract

Isosorbide (ISB), a nontoxic bio-based bicyclic diol composed from two fuzed furans, was incorporated into the preparation of flexible polyurethane foams (FPUFs) for use as a cell opener and to impart antioxidant properties to the resulting foam. A novel method for cell opening was designed based on the anticipated reversibility of the urethane linkages formed by ISB with isocyanate. FPUFs containing various amounts of ISB (up to 5 wt%) were successfully prepared without any noticeable deterioration in the appearance and physical properties of the resulting foams. The air permeability of these resulting FPUFs was increased and this could be further improved by thermal treatment at 160 °C. The urethane units based on ISB enabled cell window opening, as anticipated, through the reversible urethane linkage. The ISB-containing FPUFs also demonstrated better antioxidant activity by impeding discoloration. Thus, ISB, a nontoxic, bio-based diol, can be a valuable raw material (or additive) for eco-friendly FPUFs without seriously compromising the physical properties of these FPUFs.

## 1. Introduction

Polyurethane foams (PUFs) are versatile plastics that have many advantages over other types of foams, such as ease of processing, low density, and excellent physical properties [1]. Among PUFs, flexible PUFs (FPUFs) that have an open cell structure possess excellent air permeability and the physical properties of FPUFs, such as density and resilience, are easily controllable by varying the polyurethane formulation recipe [1,2,3]. Thus, FPUFs are widely used in many different industries, such as those making cushions for furniture and automobiles, sound-absorbing materials, and packaging materials [4,5,6,7,8,9,10]. PUFs are commonly manufactured according to the following steps: (1) Mixing of polymer components with blowing agents; (2) nucleation and growth of cells; (3) gelation and crosslinking; and (4) cell opening and curing [11,12,13]. The opening of cell windows greatly affects the air permeability of FPUFs and also imparts various physical properties to the FPUFs for a variety of applications. Cell opening is typically induced through a combination of internal and external parameters. Internal parameters include the viscosity of the liquid resin [14,15], urea precipitation [16,17], catalyst balance [13,18,19], the effect of surfactant on bubble nucleation and stability [20,21], and the addition of fillers or additives to facilitate cell opening [22,23,24,25]. External parameters enable cell opening after foaming through physical or chemical treatment, such as crushing and reticulation [1,24,26,27,28]. Furthermore, it is critical that the cell structure and walls corresponding to the polymer matrix must be robust enough to withstand the harsh processes involved in cell opening.

With increasing concerns over environmental issues and the depletion of petroleum-based raw materials, the manufacturing of products based on environmentally-friendly raw material sources, without negatively affecting the performance of the final product, is becoming increasingly important [29,30]. Accordingly, interest has grown dramatically in bio-based materials derived from natural resources that are nontoxic to humans and environmentally friendly [29,31,32,33,34]. 1,4:3,6-dianhydrosorbitol or isosorbide (ISB) is one such bio-based resource that can be derived from a natural product. ISB is manufactured via the dehydration of D-sorbitol which can be obtained by hydration of D-glucose [35,36,37,38,39]. ISB is a bicyclic diol composed of tetrahydrofuran rings and hydroxyl groups at carbons 2 and 5. The hydroxyl groups in ISB can be found in two distinct orientations, *exo and endo*, and can be easily modified according to the desired applications [40,41]. Moreover, ISB has been attracting significant interest from multiple fields because of its unique rigid bicyclic structure, nontoxicity, and its ability to improve the heat resistance and mechanical properties of polymers [38,42,43,44]. For example, ISB can be used to replace bisphenol-A for the manufacture of polycarbonates with properties of high mechanical strength and also to impart ultraviolet (UV)-resistance to polymers [43,45]. Thus, ISB can be a substitute for the role of an aromatic diol due to its bulky and rigid structure.

**Scheme 1:**
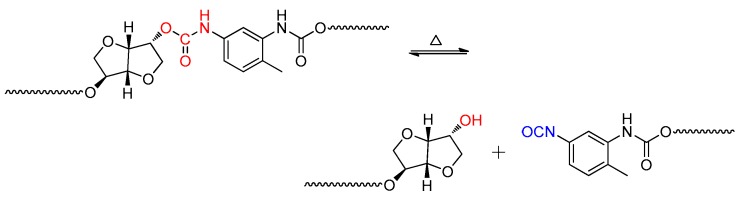
Thermal reversibility of a urethane linkage formed by the reaction between ISB and an aromatic diisocyanate.

It is well-known that the urethane units formed by the reaction between the active hydrogen in hydroxyl and isocyanate groups are reversible between 150 and 200 °C, and this feature can induce easier dissociation at the lower temperatures as the steric hindrance of both the hydroxyl groups and isocyanate groups increases [46,47,48,49,50]. In particular, phenolic hydroxyl groups can be used as a general blocking agent by reacting with the isocyanate groups to form a phenolic urethane in order to improve the storage stability of isocyanates from attack of moisture and oxygen [49,50]. At elevated temperatures, this bond can dissociate back to phenolic hydroxyl groups (blocking agent) and isocyanate groups and the regenerated isocyanate groups can participate in further polymerization reactions with hydroxyl or amine groups to form a thermally stable urethane or urea linkages. ISB, which has heterocyclic rings and two secondary hydroxyl groups, is structurally similar to the phenolic hydroxyl group. Thus, we expect that the hydroxyl groups can react reversibly with isocyanate groups to form urethane units that can be dissociated at specific temperatures. Accordingly, in this study, we proposed a novel process for the cell opening of FPUFs via a reversible urethane formation reaction between the hydroxyl groups in ISB and the aromatic isocyanate groups (Scheme 1). Thus, effects of isosorbide incorporation into the formulations of FPUFs on the foam properties were investigated. The core temperature of FPUFs typically rises to around 140–160 °C during foaming, which is sufficient to trigger urethane dissociation for units containing ISB [11,51]. As the high core temperature is reached, the dissociation of ISB-based urethane units causes a decrease in the molecular weight and modulus of polymers, and one can then expect that the relatively thin cell window layers will be broken for cell opening. The free isocyanate groups generated by the reversible urethane linkages can react with adjacent free hydroxyl groups to form urethane linkages. The free isocyanate groups may react with adjacent –NH in urethane or urea groups to form allophanate or biuret groups, respectively [49].

Another interesting observation is that FPUFs based on ISB have been shown to possess better antioxidant activity, as compared with FPUFs without ISB. In general, additives such as UV stabilizers, UV absorbers, and free radical scavengers are added in the formulation of PUFs to prevent discoloration and yellowing through oxidation [52,53]. Thus, imparting innate antioxidant activity is also important for FPUFs and this paper demonstrates that ISB can play the role of antioxidant as well as that of cell opener.

In this study, ISB was incorporated to FPUFs by dissolution in conventional poly (propylene glycol) (PPG), which enabled stable foaming during FPUF formation without deformation, shrinkage, or collapse. We investigated the effects of ISB on the air permeability, resilience, and thermal and mechanical properties of FPUFs. To the best of our knowledge, there are no other studies to date on the effect of ISB as a cell opener and antioxidant in polymers, especially FPUFs. We will thus demonstrate that ISB can be considered an attractive bio-based additive for the manufacturing of FPUFs.

## 2. Materials and Methods

### 2.1. Materials

ISB (molecular weight: 146.1 g/mol, hydroxyl value: 767.8 mg KOH/g) was obtained from Samyang Co., Ltd. (Dae-Jeon, Korea). Commercially available poly(propylene glycol) (PPG, TF-3000) with the number average molecular weight of 3000 g/mol and hydroxyl value of 56.1 mg KOH/g was obtained from SKC (Ul-San, Korea). Toluene diisocyanate (TDI-80, isomer ratio 2,4/2,6 = 8/2) was obtained from the OCI Company Ltd. (Gun-San, Korea). TDI-80 is one of popular diisocyanates in PU industries for beds and furniture. Silicone surfactant (Niax silicone L-580) was obtained from Momentive (Waterford, NY, USA). Both amine catalysts A-1 (70% bis[2-dimethylaminoethyl] ether in dipropylene glycol [DPG]) and 33-LV (33% triethylenediamine in DPG) were purchased from Aldrich (Yong-In, Korea). Dibutyltin dilaurate (DBTDL) from Aldrich was used to facilitate gelation. Distilled water was used as the blowing agent. PPG was dehydrated at 80 °C prior to use under vacuum. All chemicals were used as received.

### 2.2. Preparation of FPUFs Based on ISB

ISB was dissolved in PPG at 90 °C and then the ISB-polyol mixture was allowed to cool down to room temperature. The concentration of ISB in the polyol mixture was varied: 1; 2; 3; 4; and 5 wt%. The polyol mixtures of various ISB content were transparent and stable at room temperature (no precipitation or crystallization was observed) (Figure S1). FPUFs were prepared in two steps: Firstly, L-580 (1.20 part per hundred polyol (phr)), A-1 (0.13 phr), 33-LV (0.50 phr), DBTDL (0.16 phr), and distilled water (3.00 phr) were added to the polyol mixture containing various amounts of ISB and were mixed for 30 s; secondly, the stoichiometrically required amount of TDI-80 to react with PPG, ISB, and distilled water was quickly poured into the polyol mixture and vigorously mixed under mechanical stirring at 3000 rpm for 7 s. The isocyanate index was fixed at 100. Then the mixture was poured into a wooden open mold (200 mm × 200 mm × 200 mm) for a free rise. During the foaming, the characteristic times (cream time, rise time, and gel time) were recorded using a stopwatch according to ASTM D7487-13. Cream time (CT) is the time between the start of mixing and the point at which fine bubbles begin to appear. Rise time (RT) is the time at which the foam stops expanding, as observed visually. Gel time (GT) time at which long strings of tacky material can be pulled away from the surface of the foam when the surface is touched by the edge of a tongue depressor or similar implement. The FPUFs were demolded and cured at 110 °C for one day before characterization. After curing, visible deformations (shrinkage and collapse) were not observed (Figure S2) and the additional processes for cell opening, such as crushing and reticulation, were not performed. The samples were named PUF-IX, where X was the content of ISB in the conventional PPG mixture. Table 1 summarizes the formulation for the FPUFs with various amounts of ISB, which were designed to manufacture general purpose FPUFs for bed and furniture with density values of about 30 kg/m^3^. The bio-content in FPUFs with various ISB content is shown in Table S1.

### 2.3. Preparation of Polyurethane (PU) Films Based on ISB

To confirm the reversible feature of the urethane linkages formed between the isocyanate groups and hydroxyl groups in ISB, PU films of differing ISB content were prepared using the same components as the FPUFs, but without a silicone surfactant, amine catalyst, and blowing agent. The ISB/PPG (TF-3000) mixture was mixed with the stoichiometrically required amount of TDI-80. DBTDL at 0.1 wt% with respect to the polyol weight was added to promote gelation. Then, the mixture was degassed under vacuum and poured into a glass mold. Finally, the PU films were cured at 110 °C for 24 h. The samples were named PU-IX, where X was the content of ISB (wt%) with respect to the total PPG weight. The sample code and formulation for PU films with various ISB content are shown in Table S2.

### 2.4. Free Radical Scavenging of PU Films Based on ISB

The free radical scavenging activity of the PU films was evaluated according to the 2,2-diphenyl-1-picrylhydrazyl (DPPH) method [54,55,56]. Without exposure to light, 100 mg of the PU film containing ISB (5 wt%) was immersed in 3 mL of 0.3 mM DPPH solution (in methanol). The DPPH solution containing a PU film without ISB was also prepared as a control for comparison. The absorbance of the DPPH solution was measured at 515 nm on a Jasco V-670 (Easton, MD, USA) UV-vis spectrometer. The decrease in the absorbance at this wavelength was monitored hourly. The percentage of free radical scavenging activity was calculated using the following Equation (1):

Free Radical Scavenging Activity (%) = [(A_b_ − A_s_)/A_b_] × 100 (1)

where A_b_ is the absorbance of blank DPPH solution without PU film and A_s_ is the absorbance of the DPPH solution containing PU films.

### 2.5. Characterization

Reversibility of urethane linkages was monitored using a Jasco Fourier-transform infrared (FTIR) spectrometer (FTIR 2000, Easton, MD, USA) equipped with a heating block, thermocouple, and digital thermal controller. The PU films were dissolved in dimethylformamide and the solution was coated on a KBr window. The solvent was then removed with mild heating (60 °C). FTIR spectra were recorded in the wave number range from 4000 to 500 cm^−1^ at a resolution of 4 cm^−1^. SEM was performed on a Jeol JSM 6400 (Akishima, Tokyo, Japan) to examine the cell morphology at an accelerating voltage of 20 kV. The samples were prepared by coating with gold to avoid charging of the electrons before measurement. The average cell size and thickness of cell walls were determined by using analysis software. About 60–70 cells per each sample were counted to determine the average cell size and thickness of cell walls. Air permeability of the FPUFs was evaluated using a Hallam F0023 foam porosity tester (Victoria, Australia), according to ASTM D 3574. The test specimens were cut into 50 mm × 50 mm × 25 mm (width × length × height) pieces and placed in the test cavity. The pressure differential was controlled at 125 Pa and it was maintained for 10 s. The tensile properties of the FPUFs, such as tensile stress and elongation at break, were measured using a universal testing machine (UTM, Z020, Zwick/Roell, Ulm, Germany). Dog bone specimens (10 mm thick) were used for the measurements and the crosshead speed were set at 500 mm/min. The tensile tests of four specimens per sample were evaluated and averaged. Tear strength was evaluated using a UTM and the procedure for tear strength testing was identical to that of the tensile test. Compression force deflection (CFD) was measured using UTM (Z020, Zwick/Roell, Ulm, Germany) according to ASTM D 3574. Three specimens per samples were measured and averaged. The resilience of FPUFs was evaluated by employing a ball rebounding tester according to ASTM D 3574. The center of the test specimens (100 mm × 100 mm × 50 mm; width × length × height) was located at the bottom center of the tube. Then, a 16.3 g steel ball (16 mm diameter) was dropped from a height of 500 mm and the maximum rebound height was recorded. The median of three specimens per sample was obtained. The thermal decomposition behavior of the FPUFs was investigated by employing TGA from TA instruments (Q50 machine, New Castle, DE, USA). Small pieces of sample were placed on a platinum pan and heated from room temperature to 800 °C at a heating rate of 20 °C/min under nitrogen. TGA measurements were performed thrice per sample and representative data were used for analysis. Dynamic mechanical property measurements of the FPUFs were carried out on a DMA from TA instruments (Q800, New Castle, DE, USA) in tension mode from −100 °C to 150 °C at a heating rate of 5 °C/min (frequency of 1 Hz and amplitude of 15%).

## 3. Results and Discussion

### 3.1. Physical Properties of FPUFs Based on ISB

For all the FPUFs in this study, the same amounts of catalyst and blowing agent were added during formulation. Thus, the changes in reactivity and density are entirely dependent on ISB content and the effects of ISB. ISB was successfully incorporated into FPUFs, and these FPUFs formed open cellular structures without any deterioration (shrinkage or collapse). Table 2 shows the reactivity and density of FPUFs formed with differing amounts of ISB. The CT was found to increase with increasing ISB content. In the formulations of FPUFs, the amount of TDI-80 increased with increasing ISB content due to the increased hydroxyl value of polyols, while the catalyst content was fixed. Thus, the concentrations of the catalysts in the formulations were lowered with increasing the ISB content. The increase of CT with increasing ISB content is attributable to the decrease of catalysts concentrations as well as the increased viscosity of the polyol/ISB mixture (Table S3). This increased difficulty in reactive contact of the reagent molecules and hampered the blowing reaction. Accordingly, FPUFs based on ISB exhibited a slower RT, as compared with the control FPUF (PUF-I0). FPUFs with less ISB content (PUF-I1 and PUF-I2) showed an increased GT; however, GT decreased with increasing ISB content. This was mainly due to the increased number of hydroxyls in the polyol/ISB mixture because there are more hydroxyl groups that are capable of reacting with isocyanate groups in the overall mixture. Thus, more urethane bonds could be formed by the incorporation of ISB. Furthermore, the hydroxyl groups of the short diol, ISB, can approach isocyanate groups more easily than those hydroxyl groups of conventional PPG, leading to a decrease in GT. Density was an important parameter in the physical properties of FPUFs and it strongly depended on the amount of blowing agent [13]. Since the same amount of distilled water was used for the preparation of all FPUFs, all the FPUFs had a similar density (30 ± 1 kg/m^3^). This indicated that the incorporation of ISB did not influence the density of FPUF.

**Figure 1:**
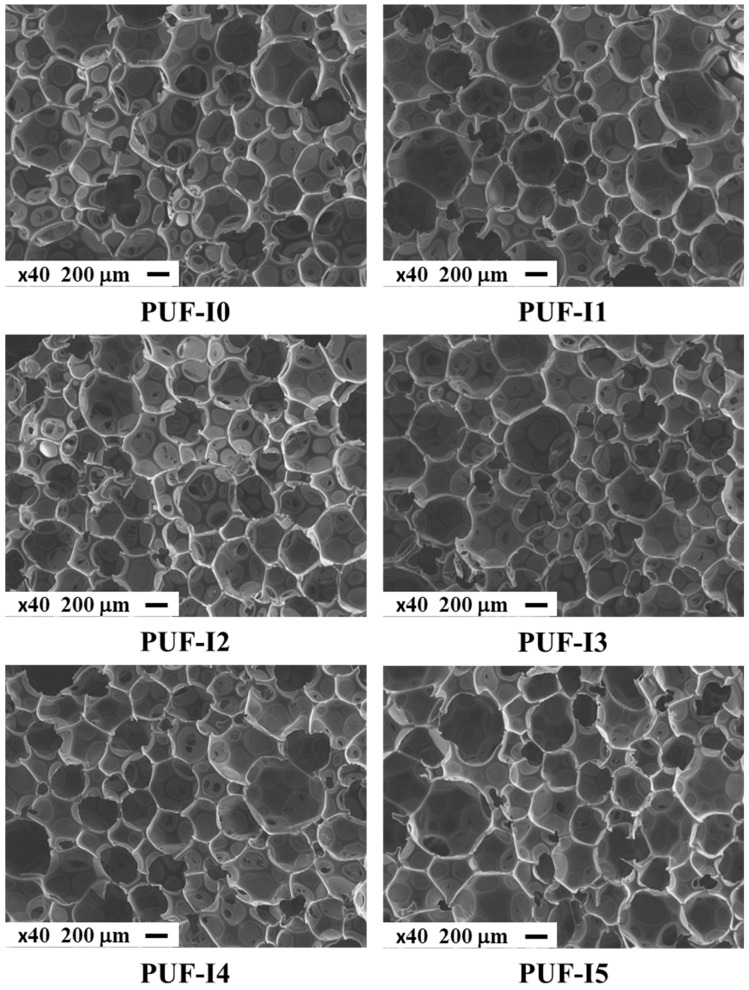
SEM images of the various FPUFs studied.

**Figure 2:**
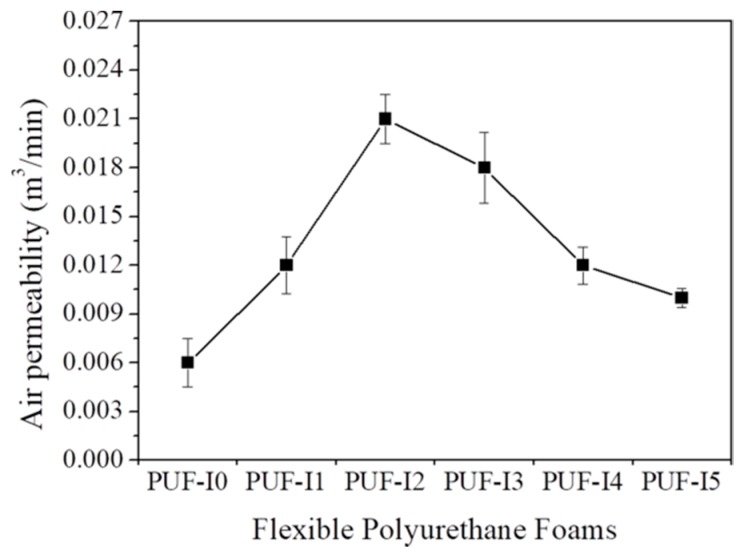
Air permeability of the various FPUFs studied.

Figure 1 shows the scanning electron microscopy (SEM) images of the various FPUFs studied. All the FPUFs showed spherical or polyhedral shapes. Broken and contorted cells were not observed; thus, incorporation of ISB did not lead to such negative effects. Average cell size, average thickness of cell walls, and the number of cells per unit area of ISB-containing FPUFs are summarized in Table S4. The incorporation of ISB to FPUFs led to a slight decrease in average thickness of cell walls due to the slower CT. That is, slower CT implies the slow increase in mixture viscosity allowing the liquid layer between the bubbles to thin during initial cell formation. On the other hand, the average cell size of FPUFs containing ISB was reduced in comparison with that of control FPUF (PUF-I0) due to the decreased GT. Thus, it was observed that the number of cells of ISB-containing FPUFs per unit area were larger than that of control FPUF. The fast formation of urethane or urea linkage may restrict the growth and coalescence of bubbles, and this leads to the formation of smaller cell sizes. Some of the cell windows were also found partially open but most of them were closed. In this study, the FPUFs did not allow any physical and chemical treatment after their preparation to confirm the effect of ISB as a cell opener. The air permeability of FPUFs was investigated to quantitatively evaluate the degree of cell opening and the results are shown in Figure 2. The air permeability of the FPUFs increased with increasing ISB content. The maximum air permeability was found for 2 wt% ISB content (0.021 m^3^/min). This was nearly three times that of the control FPUF (0.006 m^3^/min). Further increases in ISB content led to decreased air permeability due increased formation of closed cells. In general, closed cells can be formed when the gelation dominates the blowing reaction during formation of FPUFs. As shown in Table 2, the incorporation of ISB slowed the blowing reaction and accelerated the gelation reaction. Accordingly, the air permeability of FPUFs with high ISB content became reduced.

**Figure 3:**
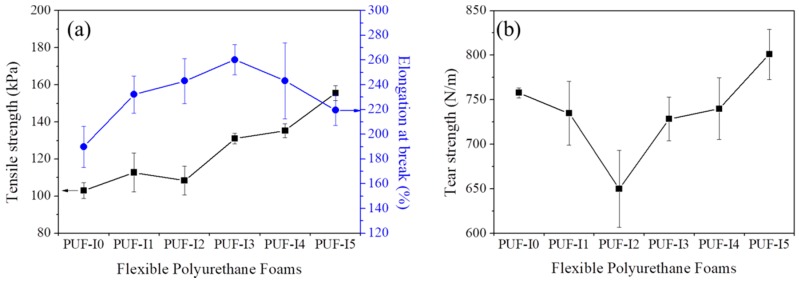
(**a**) Tensile strength and elongation at break; and (**b**) tear strength of the various FPUFs.

ISB contains a fuzed bicyclic furan structure; thus, it can impart stiff properties and lead to a high glass-transition temperature [38]. Another factor for concern is that FPUFs based on ISB might exhibit a lower crosslinking density. Because ISB has two hydroxyl groups, the incorporation of ISB into FPUFs leads to a longer distance between the physical crosslinking points (Figure S3). Therefore, the addition of ISB may have both a reinforcing effect (from the fused bicyclic furan) and a plasticizing effect (by lowering the crosslinking density). Figure 3a shows the tensile properties of the FPUFs with various ISB content. The tensile strength of the FPUFs based on ISB was enhanced compared with the control FPUF (103.0 kPa). Tensile strength was found to increase with increasing ISB content and the maximum was 155.6 kPa for PUF-I5. When 2 wt% ISB was incorporated into FPUF, a slight decrease in the tensile strength (108.4 kPa) was observed, which was attributed to the higher air permeability. In general, the mechanical properties of FPUFs weaken as the open cell content increases [4,57]. FPUFs based on ISB also showed an increased elongation at break compared with the control FPUF, which was attributed to decrease in crosslinking density of the polymer network. When a high content of ISB was incorporated (PUF-I4 and PUF-I5), a decrease in the elongation was observed at break due to the increased stiffness.

Figure 3b shows the tear strength of the various FPUFs. The tear strength of FPUFs based on ISB was comparable or slightly improved over that of the control FPUF. The observed decrease and thus weakening in tear strength for PUF-I2 was caused by the increase in open cell windows. Furthermore, ISB-containing FPUFs prepared in this study exhibited the comparable or superior tensile and tear strength to reference FPUFs in the literature (100–150 kPa for tensile strength and 500–880 N/m for tear strength) [1,2,3,27].

**Figure 4:**
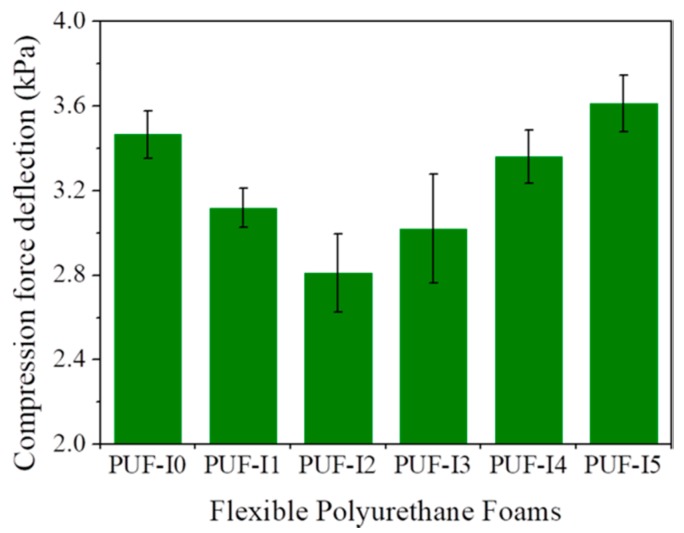
CFD of FPUFs with various ISB content.

Figure 4 shows the CFD of the FPUFs with various ISB content. CFDs of FPUFs with lower ISB content (PUF-I1 and PUF-I2) were lower than that of PUF-I0 due to the increased air permeability. It is speculated that the increase of cell opening led to the decrease in resistance to external compression force. Further increase of ISB content led to an increase of CFD, which is attributed to the increased stiffness by the incorporation of ISB as well as decreased air permeability (increased closed cell content). The slightly enhanced CFD value of PUF-I5 (3.61 kPa) was observed compared with PUF-I0. However, CFD of ISB-based FPUFs prepared in this study showed a lower value compared with that of reference FPUF in the literature (>4~5 kPa) [58].

**Figure 5:**
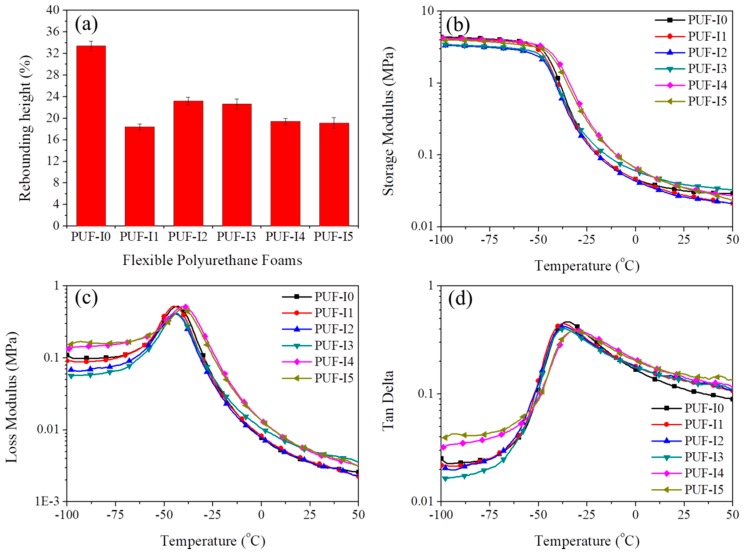
(**a**) Maximum rebounding height (%); (**b**) temperature dependence of storage modulus; (**c**) loss modulus; and (**d**) tan δ curves of the various FPUFs.

The resilience of FPUFs is another important parameter for specific FPUF applications, such as automotive seats and furniture. The resilience of FPUF is strongly affected by the molecular weight and structure of soft and hard domains, crosslinking density, fillers, and the proportion of open cells [59,60,61]. The resilience of FPUF is generally evaluated by the ball rebounding test and loss factor (tan δ) in dynamic mechanical analyzer (DMA) measurement. The maximum ball rebound height of the prepared FPUFs is presented in Figure 5a. All the ISB containing FPUFs exhibited a lower rebounding height than the control FPUF (PUF-I0). This was attributed to the decreased crosslinking density from ISB incorporation [59]. However, ball rebounding height of ISB-containing FPUFs did not show significant trend with ISB content. Figure 5b–d show the DMA results (storage modulus, loss modulus, and tan δ curves) of the various FPUFs. The glass-transition temperature (T_g_) was determined as the maximum point in the tan δ curve, and the results are summarized in Table 3. The storage moduli of the FPUFs with less ISB content (PUF-I1 and PUF-I2) at room temperature were lower than the control FPUF. This was mainly due to the loosening of the crosslinking network caused by the lengthened physical crosslinking points; thus, the T_g_s of ISB-containing FPUFs were reduced (Table 3). On the other hand, further increase in ISB content led to an increase in the storage modulus and T_g_ again, resulting from the increased stiffness by the addition of ISB with rigid bicyclic ring. The peak value and width of the tan δ curves is another meaningful indicator of resilient performance of FPUFs [25,61]. As shown in Figure 5d, the tan δ values of the ISB-containing FPUFs were higher compared with the PUF-I0 at room temperature; therefore, a decreased resilience can be expected for the ISB-containing FPUFs. In common with ball rebounding test, the significant change in resilience performance of ISB-containing FPUFs was not found to depend on ISB content. It indicates that resilient performance of FPUFs was reduced by the incorporation of ISB, but no significantly affect on ISB content.

### 3.2. Cell Opening by the Reversibility of Urethane Units Based on ISB in FPUFs

**Figure 6:**
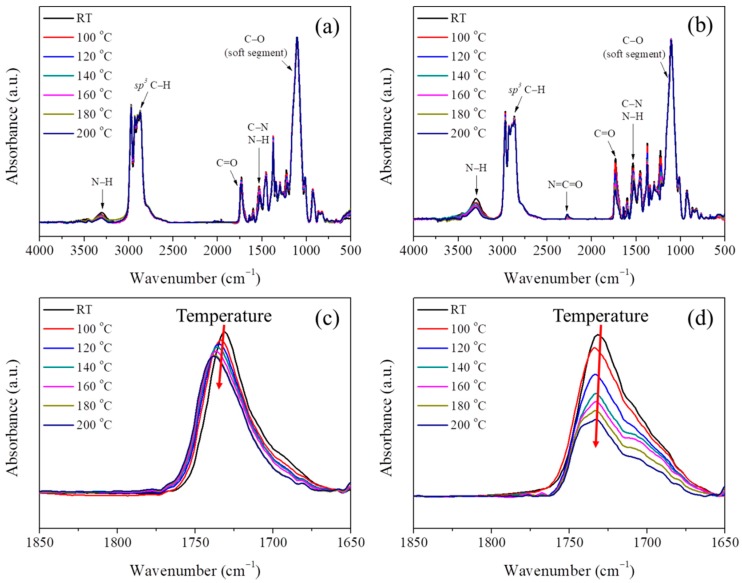
Temperature-dependent FTIR spectra of PU films: (**a**) PU-I0; (**b**) PU-I5; (**c**) PU-I0 expanded at 1650–1850 cm^−1^; and (**d**) PU-I5 expanded at 1650–1850 cm^−1^.

We studied the effect of ISB as a thermally triggered reversible cell opening agent (Scheme 1). In general, the core temperature of the FPUF during foaming of slab stocks in the plants approaches 160 °C due to the exothermic heat of reaction [11]. This temperature is sufficient for urethane bonds formed by ISB in the polymer network to form and break reversibly. At that moment, the modulus of polymers, especially for thin cell windows, weakens and then the weak cell windows can be opened. One important consideration for FPUFs is that the solid matrix that forms the cell strut and wall should be strong enough to withstand the harsh cell opening conditions. If the solid matrix is too weak, the cell walls break and the overall structure can even collapse, leading to serious deterioration of the mechanical properties of the FPUFs. In this experimental study, the foam dimensions prepared in the laboratory were too small for the temperature to reach 160 °C during the foaming. While the air permeability of the ISB-containing FPUFs was improved, as compared with the control FPUF (Figure 2), the ISB-containing FPUFs were thermally treated in a convection oven (160 °C) to confirm the desired effect of ISB as a cell opener via thermal reversibility of the urethane linkages. Before thermal treatment, the reversibility of the urethane linkages formed by ISB and isocyanate groups was confirmed using temperature-variable FTIR. It is difficult to evaluate the reversibility of urethane bonds in a foam, thus we prepared PU films using the same raw materials but without surfactant, catalyst, and distilled water (see experimental section for details). The chemical compositions of PU films were different from those of FPUFs since PU films were prepared in the absence of distilled water; thus they did not contain urea linkages. According to Delebe and Rolph, the urea linkages are more stable than urethane linkages thermally [47,48]. Temperature-dependent FTIR spectra of PUF-I0 and PUF-I5 are shown in Figure S4. When the temperature was increased above 160 °C, the peak intensity of urea carbonyl groups (C=O) at 1690 cm^−1^ decreased significantly in both FTIR spectra of PUF-I0 and PUF-I5, as shown in Figure S4c,d. Accordingly, peaks due to the isocyanate groups could also be observed at 2270 cm^−1^ (Figure S4e,f). The generation of isocyanate groups for PUF-I0 was attributed to the thermal reversibility of urea bonds, while that for PUF-I5 was due to the thermal reversibility of both urethane and urea bonds. The isocyanate peak in FTIR of PUF-I0 was observed at higher temperature (180 °C) compared with PUF-I5 (160 °C), implying that the reversibility of ISB-based urethane linkages appeared at lower temperature. Consequently, the PU films without urea linkages would be the better choice to confirm the reversibility of ISB-based urethane linkages. Figure 6 shows the FTIR spectra of PU films with or without ISB at different temperatures. To facilitate comparison of the temperature-dependent changes, all the FTIR spectra were normalized against the intensity of the C–O peak in the soft segments (1110 cm^−1^) [5,62,63]. The absorption peak of the isocyanate group can be observed at 2270 cm^−1^. An expansion of the FTIR spectra near the characteristic peak of the isocyanate group for the PU films with differing ISB content is shown in Figure S5. No isocyanate peak was observed across the entire temperature range for PU films without ISB (PU-I0). However, the PU film with 5 wt% ISB showed a clear absorption peak near 2270 cm^−1^, corresponding with the isocyanate group when the temperature exceeded 160 °C (Figure S5). Furthermore, while PU-I0 showed a slight decrease in peak intensity at 1730 cm^−1^ (assigned to the absorption of carbonyl of urethane groups) as the temperature changed, PU-I5 showed a large decrease in the peak intensity with increasing temperature. The decrease of peak intensity at 1730 cm^−1^ is attributable to the reversible urethane groups. Therefore, the reversibility of urethane groups at elevated temperatures (at least 160 °C) was confirmed for ISB-containing PU films and cell opening thermal treatment should be performed above 160 °C.

**Figure 7:**
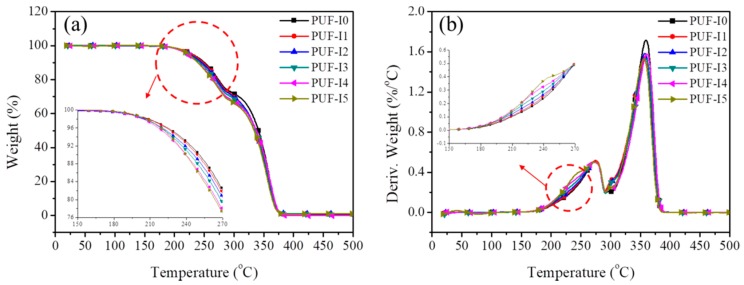
TG (**a**) and DTG (**b**) thermograms of FPUFs containing various amounts of ISB in nitrogen gas atmosphere.

The thermal reversibility of ISB-based urethane units was also confirmed by thermogravimetric analysis (TGA) measurements. Figure 7 shows the thermogravimetry (TG) and derivative TG (DTG) thermograms measured under nitrogen atmosphere. The TG and DTG curves revealed two main decomposition stages. The first stage occurs at 270 °C and corresponds to the dissociation and break down of the hard segments. The second stage occurs at 360 °C and is associated with the decomposition of the soft segments; that is, conventional PPG [30,64]. The characteristic temperatures of various FPUFs obtained from TGA measurement were summarized in Table 4. The two maximum decomposition temperatures did not show significant changes depending on ISB content. All the FPUFs exhibited the almost similar maximum decomposition temperature of soft and hard segments. One remarkable observation was that the shoulder at about 240 °C in the decomposition of the hard segments became increasingly prominent as the ISB content increased. This shoulder was not observed in PUF-I0, but it could be evidently observed for the FPUFs containing ISB. To determine what this shoulder peak indicated, we scrutinized our FTIR data. Both hydroxyl groups and isocyanate groups were completely consumed during the preparation of the FPUFs (Figure S6). No peaks corresponding to the free hydroxyl (3400–3600 cm^−1^) or the isocyanate functional groups (2270 cm^−1^) were observed in the FTIR spectra of any of the FPUFs studied. Therefore, the shoulder peak is strongly believed to be the decomposition of hard segments specific to the reversible urethane linkages formed between the hydroxyl groups of ISB and the isocyanate groups. Accordingly, T_5%_ and T_10%_ were reduced with increasing ISB content due to the thermal decomposition of ISB, resulting from the reversibility of ISB-based urethane linkages at elevated temperature.

**Figure 8:**
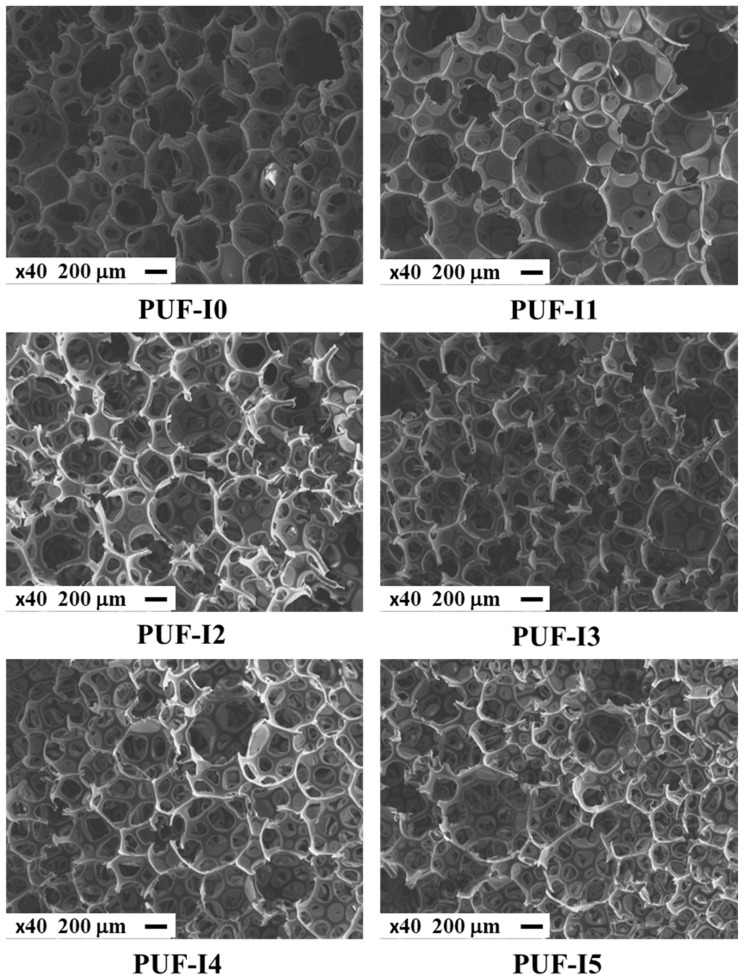
SEM images of the FPUFs after thermal treatment at 160 °C.

**Figure 9:**
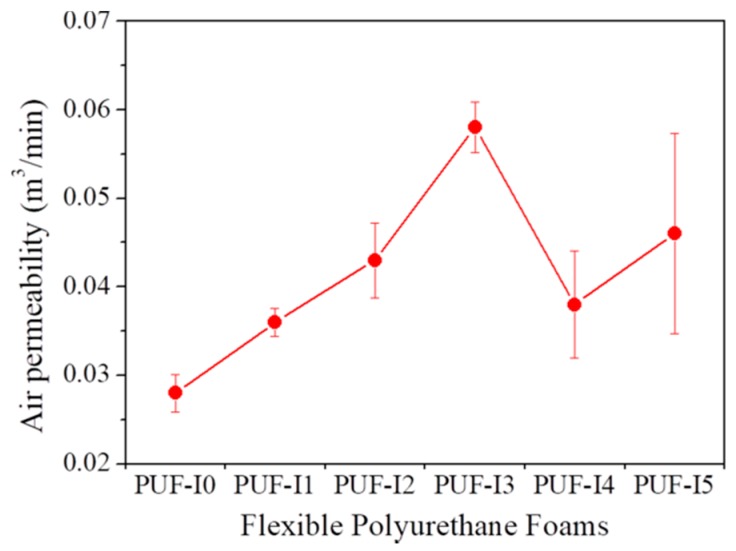
Air permeability of the FPUFs with various ISB content after thermal treatment at 160 °C.

SEM images of the FPUFs after thermal treatment at 160 °C are presented in Figure 8. The average cell size and average thickness of cell walls are summarized in Table S4. The average thickness of cell walls of FPUFs after thermal treatment was not significantly changed compared with those of FPUFs before thermal treatment. However, the average cell size was slightly reduced after thermal treatment. When compared with the SEM images of FPUFs before thermal treatment (Figure 1), almost all of the cell windows in the ISB-containing FPUFs were opened without serious destruction of the cell wall and of the overall structure. Furthermore, the opening of the cell windows occurred more noticeably for the FPUFs with higher ISB content. This indicates that cell opening can occur through the designed reversible urethane linkages formed by the hydroxyl groups of ISB and the isocyanate groups. Figure 9 shows the air permeability of the studied FPUFs after thermal treatment at 160 °C. The air permeability data revealed that the air permeability of FPUFs based on ISB increased significantly, in good agreement with the SEM images. PUF-I0 also exhibited increased air permeability after thermal treatment (0.028 m^3^/min), indicating that thermal treatment itself causes cell opening for even FPUFs without ISB. However, the ISB-containing FPUFs showed better air permeability as compared with PUF-I0, suggesting that the designed reversible urethane linkages based on ISB can additionally weaken the modulus of the polymer to further promote the opening of cell windows. The maximum air permeability was obtained for the FPUF with 3 wt% ISB after the thermal treatment.

### 3.3. Antioxidant Activity of ISB-containing FPUFs

**Figure 10:**
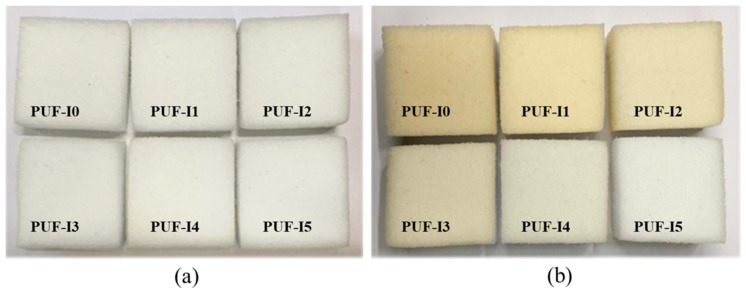
Color comparison of FPUF samples (**a**) before and (**b**) after thermal treatment at 160 °C.

Interestingly, we found that the difference in yellowing was significant for the ISB-containing FPUFs after thermal treatment at 160 °C. In general, polymers or plastics containing aromatic rings exhibit yellowing by atmospheric oxygen, light, and heat, causing change from the original color to yellow (yellowing). As is shown in Figure 10, all the FPUFs were bright beige before the thermal treatment. After thermal treatment, the observed discoloration was dependent on the ISB content. In other words, the control FPUF without ISB showed much yellowing after the thermal treatment at 160 °C; however, the levels of yellowing decreased as the ISB content in the FPUFs increased. Additionally, when PUF-I0 and PUF-I5 were left under ambient conditions (in air) for more than 30 days, the degree of yellowing between the two foams was noticeably different (Figure S7). The yellow index (YI) of the FPUF samples was investigated according to ASTM E313 and the results are presented in Table 5. As the content of ISB increased, the YI value of FPUFs significantly decreased. The YI value decreased from 46.40 for PUF-I0 to 19.72 for PUF-I5. Thus, the FPUFs containing ISB were demonstrated to prevent or slow FPUF oxidation caused by external factors (oxygen, light, and heat) and showed better antioxidant properties, as compared with the control FPUF without ISB. Further studies on the effects of ISB in comparison with the commercial anti-yellowing agents for FPUFs are necessary for the practical applications in industries.

**Scheme 2:**
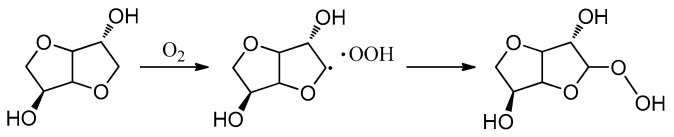
Scheme for furan ring autoxidation in ISB [67].

**Scheme 3:**
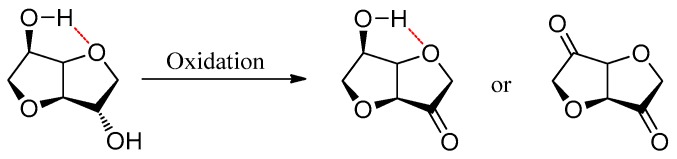
Scheme for possible hydroxyl group oxidation in ISB [69].

In general, antioxidants can possess a variety of functional groups depending on the specific starting material, which includes phenolic hydroxyls, amines, and phosphoryl groups. Specific to polymers, phenolic antioxidants or hindered amine light stabilizers are widely used as primary antioxidants to stabilize polymers or plastics from the UV-mediated oxidation by absorbing UV light or scavenging the resulting free radicals [54,65,66]. The antioxidant activity of ISB can be inferred from the following: At least some ISB may be autoxidized on exposure to atmospheric oxygen, as is shown in Scheme 2 [67]. Matsubara et al. in their study reported that tetrahydrofuran (THF) and tetrahydropyran (THP) groups can be easily oxidized at the α-position to the ring oxygen atom to form peroxides [68]. Therefore, it is postulated that ISB may prevent degradative polymer oxidation through oxygen capture or free radical scavenging. Another possibility is that the hydroxyl groups on ISB can be oxidized to form a ketone, although the oxidation of the hydroxyl group on ISB will have low levels of conversion (4%) in the absence of a catalyst (Scheme 3) [69]. The two hydroxyl groups on ISB can both be oxidized and different amounts of Gibbs free energy are required for the oxidation of these hydroxyls (compared to generic secondary alcohols), due to the higher steric hindrance of *endo*-hydroxyl groups. This means that ISB itself will demonstrate lower antioxidant activity outside of a FPUF. In the 2,2-diphenyl-1-picrylhydrazyl (DPPH) test of ISB, the free radical scavenging activity of the pure ISB solution (0.3 mM in methanol) showed a lower value (5.1%) than the equivalent value for ISB-containing PU (Figure S8).

**Figure 11:**
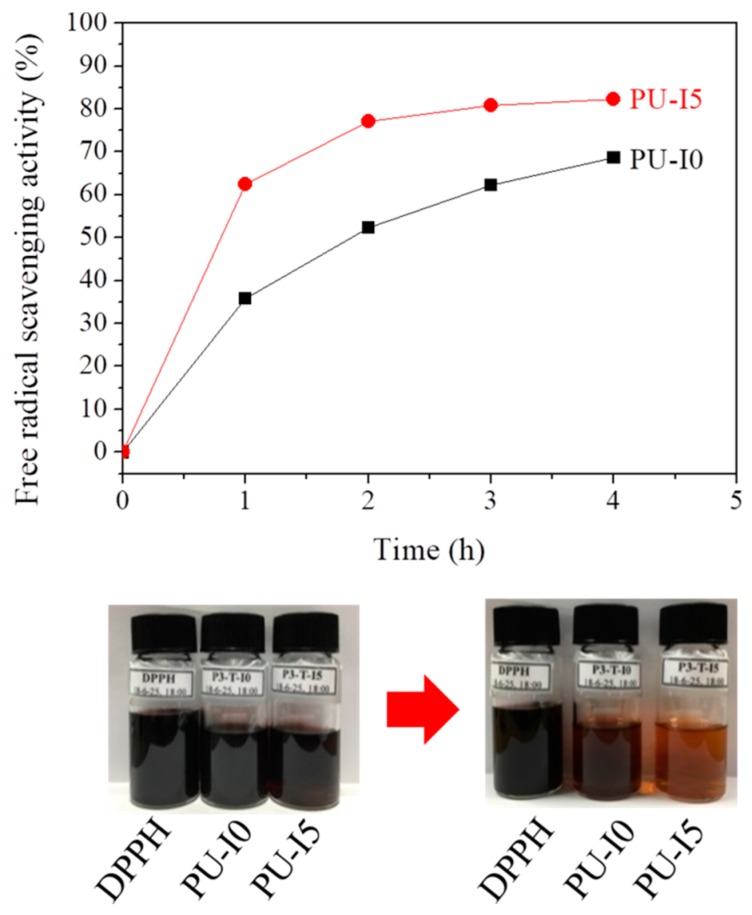
Free radical scavenging activity of PU films with or without ISB.

As shown in Scheme 2 and Scheme 3, free radicals formed in ISB molecules can act as a radical scavenger to terminate the chain reaction of free radicals and stabilize the polymers from oxidation. Accordingly, free radical scavenging activity of ISB-containing PU films was evaluated by using the DPPH method, which is widely used for evaluation of the antioxidant activities of polymers [55,56]. Figure 11 shows free radical scavenging activity of PU samples with or without ISB, and this test was performed by using PU films containing ISB instead of FPUFs as the foams absorbed the DPPH solution. The representative PU films with 0 and 5 wt% ISB of polyols (PU-I0 and PU-I5, respectively), were tested. The blank DPPH solution without sample showed almost constant absorbance over time while the DPPH solution containing PU films showed a decrease in absorbance over time at 515 nm. Because conventional PPG for FPUF contains an antioxidant in general, the absorbance of the ISB-free PU film (PU-I0) decreased. However, a far greater decrease in absorbance was observed for PU-I5. After immersion time of 4 h, the free radical scavenging activity calculated using Equation (1) was 68.7% and 82.3% for the PU film free of and the PU film containing ISB, respectively, which indicated that the ISB-containing PU film possesses better antioxidant activity. For future work, more experiments and analyses are needed to elucidate the antioxidant mechanism of ISB.

## 4. Conclusions

The rigid bicyclic diol, ISB, could be successfully incorporated into the FPUFs. ISB was miscible with conventional PPG up to 5 wt% of polyols, and the effects of ISB as a cell opener on the FPUFs were investigated. The maximum improvement in air permeability of FPUFs was observed in FPUF with 2 wt% of ISB in polyol. The slight decrease in air permeability of ISB-based FPUFs with further increase of ISB content above 2wt% was attributed to the increase of gelation rate, hindering the cell opening of FPUFs. The FPUFs based on ISB also exhibited comparable or superior mechanical properties (tensile strength and tear strength) in comparison with the control FPUF without ISB (PUF-I0) due to the inherent nature of ISB having a rigid and stiff bicyclic structure. Cell opening of ISB-containing FPUFs was studied additionally by heating to 160 °C, the core temperature of FPUFs during the foaming in the plants for the slab stock production. The air permeability of the FPUFs after the thermal treatment was significantly improved; in particular, the ISB-containing FPUFs showed 100% increases in air permeability compared with the control FPUF. Thus, the reversibility of the urethane linkages can be helpful for decreasing the modulus of the polymer at elevated temperatures and for promoting the cell opening.

Interestingly, the FPUFs containing ISB exhibited excellent antioxidant activity. In the DPPH test, the ISB-containing PU film showed increased free radical scavenging activity (up to 82.3%) compared with the ISB-free control (62.5%), indicating the better antioxidant activity of the ISB-based PU polymer. In addition, the yellow index of PUF-I5 was significantly lower than that of PUF-I0 after thermal treatment at 160 °C. Consequently, ISB is a promising bio-based raw material or additive for FPUFs as both an antioxidant and a cell opener without serious deterioration on the physical properties of FPUFs.

## Figures and Tables

**Table 1:** Sample code and formulation for FPUFs with various amounts of ISB.

Sample Code	PUF-I0	PUF-I1	PUF-I2	PUF-I3	PUF-I4	PUF-I5
	(Composition by wt.)					
***Polyol part***						
TF-3000	100	99.0	98.0	97.0	96.0	95.0
ISB	-	1.00	2.00	3.00	4.00	5.00
L-580	1.20	1.20	1.20	1.20	1.20	1.20
A-1	0.13	0.13	0.13	0.13	0.13	0.13
33-LV	0.50	0.50	0.50	0.50	0.50	0.50
DBTDL	0.16	0.16	0.16	0.16	0.16	0.16
Distilled water	3.00	3.00	3.00	3.00	3.00	3.00
***Isocyanate part***						
TDI-80	37.7	38.8	39.9	41.0	42.1	43.2
**Isocyanate index**	100	100	100	100	100	100

**Table 2:** Characteristics of FPUFs with differing amounts of ISB.

Sample Code	PUF-I0	PUF-I1	PUF-I2	PUF-I3	PUF-I4	PUF-I5
CT (s)	11	11	13	14	16	17
RT (s)	72	79	79	76	76	76
GT (s)	93	101	98	90	86	76
Density (kg/m^3^)	31	31	30	29	30	31

**Table 3:** Glass transition temperature (T_g_) of the various FPUFs determined by DMA.

Sample Code	PUF-I0	PUF-I1	PUF-I2	PUF-I3	PUF-I4	PUF-I5
T_g_ (°C)	−36.7	−37.8	−37.7	−37.4	−31.4	−31.2

**Table 4:** Characteristic decomposition temperatures of various FPUF.

Sample Code	PUF-I0	PUF-I1	PUF-I2	PUF-I3	PUF-I4	PUF-I5
T_5%_^(a)^ (°C)	230.2	228.5	225.3	223.7	221.3	221.3
T_10%_^(b)^ (°C)	250.2	248.6	246.2	243.0	239.5	238.2
T_max1_^(c)^ (°C)	273.3	274.3	274.3	274.3	273.3	274.3
T_max2_^(d)^ (°C)	359.7	357.8	357.8	358.7	360.5	355.8

^(a)^ T_5%_ indicates the decomposition temperature at 5 wt% of weight loss; ^(b)^ T_10%_ indicates the decomposition temperature at 10 wt% of weight loss; ^(c)^ T_max1_ indicates the maximum decomposition temperature of first decomposition stage; and ^(d)^ T_max2_ indicates the maximum decomposition temperature of second decomposition stage.

**Table 5:** YI of the FPUFs after thermal treatment at 160 °C.

Sample Code	PUF-I0	PUF-I1	PUF-I2	PUF-I3	PUF-I4	PUF-I5
YI	46.4	45.3	35.0	29.2	23.6	19.7

## Supplementary Materials

Figure S1: Photographs of TF-3000 (PPG)/ISB mixture samples containing different amount of ISB. The last numbers of the sample codes denote wt% of ISB in the samples, Figure S2: Photographs of FPUFs investigated in this study, Figure S3: Schematic illustration of crosslinked network structures of (a) PU without ISB and (b) those with ISB, Figure S4: Temperature-dependent FTIR spectra of PUF-I0 and PUF-I5: (a) PUF-I0; (b) PUF-I5; (c) PUF-I0 expanded at 1660–1850 cm^−1^; (d) PUF-I5 expanded at 1660–1850 cm^−1^; (e) PUF-I0 expanded at 2150–2400 cm^−1^; (f) PUF-I5 expanded at 2150–2400 cm^−1^, Figure S5: Temperature-dependent FTIR spectra of PU films based on different concentrations of ISB, Figure S6: FTIR spectra of different FPUFs after curing at room temperature for 1 h, Figure S7: Photographs of PUF-I0 (left) and PUF-I5 (right) left at room temperature for 30 days, Figure S8: Free radical scavenging activity of ISB solution in methanol (0.3 mM) by DPPH method at room temperature, Table S1: Bio-based content of FPUFs investigated, Table S2: Sample code and formulation for PU films with various ISB content, Table S3: Shear viscosity of PPG/ISB mixture at 25 °C, Table S4: Average cell size, average thickness of cell walls, and the number of cells per unit area of FPUFs with various ISB content before and after thermal treatment.
