# Supplementary material for: Effects of Isosorbide Incorporation into Flexible Polyurethane Foams: Reversible Urethane Linkages and Antioxidant Activity
Source: Molecules. 2019 Apr 5;24(7):1347. doi: 10.3390/molecules24071347 (PMC6479515; doi:10.3390/molecules24071347)
Supplement: Supplementary file 1 [file molecules-24-01347-s001.pdf]

Supplementary Materials

Effects of Isosorbide Incorporation into Flexible Polyurethane Foams: Reversible Urethane Linkages and Antioxidant Activity

Se-Ra Shin [1,†], Jing-Yu Liang [1,†], Hoon Ryu [2], Gwang-Seok Song [2] and Dai-Soo Lee [1,*]

[1] Division of Semiconductor and Chemical Engineering, Chonbuk National University, 567 Baekjedaero, Deokjin-gu, Jeonju 54896, Korea; srshin89@jbnu.ac.kr (S.-R.S.); liangjy@naver.com (J.-Y.L.)

[2] Industrial Biotechnology Program, Chemical R&D Center, Samyang Corporation, Daedeok-daero 730, Yuseong-gu, Daejeon 34055, Korea; hoon.ryu@samyang.com (H.R.); gwangseok.song@samyang.com (G.S.S.)

† Contributed equally to this work

* Correspondence: daisoolee@jbnu.ac.kr; Tel.: +82-63-270-2310

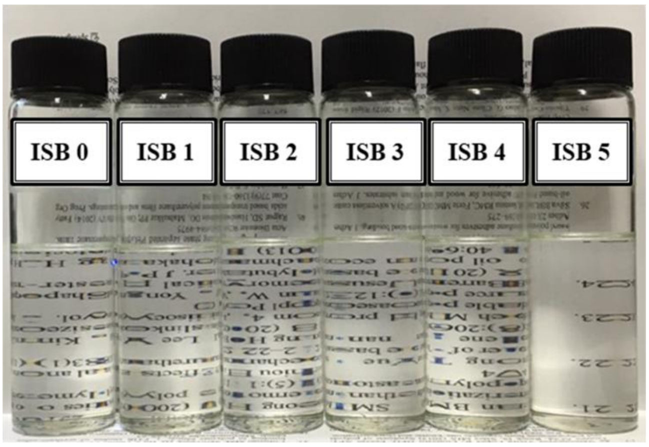

Figure S1. Photographs of TF-3000 (PPG)/ISB mixture samples containing different amount of ISB. The last numbers of the sample codes denote wt.% of ISB in the samples.

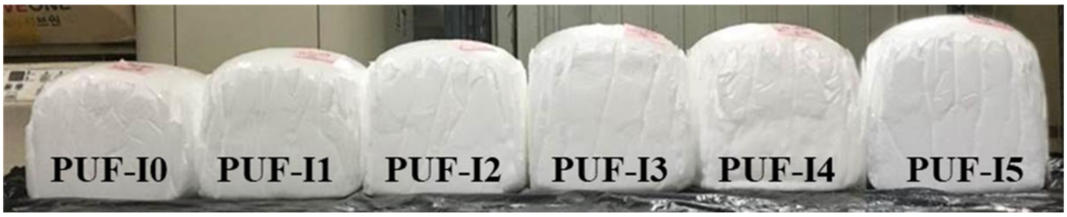

Figure S2. Photographs of FPUFs investigated in this study.

Table S1. Bio-based content of FPUFs investigated.

**Sample code**	**PUF-I0**	**PUF-I1**	**PUF-I2**	**PUF-I3**	**PUF-I4**	**PUF-I5**
Bio-content (wt%)	0	0.70	1.38	2.05	2.72	3.37

Table S2. Sample code and formulation for PU films with various ISB content.

Sample code	PU-I0	PU-I1	PU-I2	PU-I3	PU-I4	PU-I5
	(Composition by wt.)					
TF-3000	100	99.0	98.0	97.0	96.0	95.0
ISB	-	1.00	2.00	3.00	4.00	5.00
TDI-80	8.70	9.80	10.9	12.0	13.1	14.2
DBTDL	0.11	0.11	0.11	0.11	0.11	0.11
Isocyanate index	100	100	100	100	100	100

Table S3. Shear viscosity of PPG/ISB mixture at 25 °C.

Sample code*	ISB-0	ISB-1	ISB-2	ISB-3	ISB-4	ISB-5
Viscosity **(cps)	528.2	563.4	571.3	572.5	617.0	636.9

* In the sample code, ISB-X, X is the content of ISB (by wt. %) in the PPG polyol.
** Shear viscosity was determined at shear rate of 1 s^{-1} at 25 °C employing a parallel-plate rheometer from TA (AR 2000).

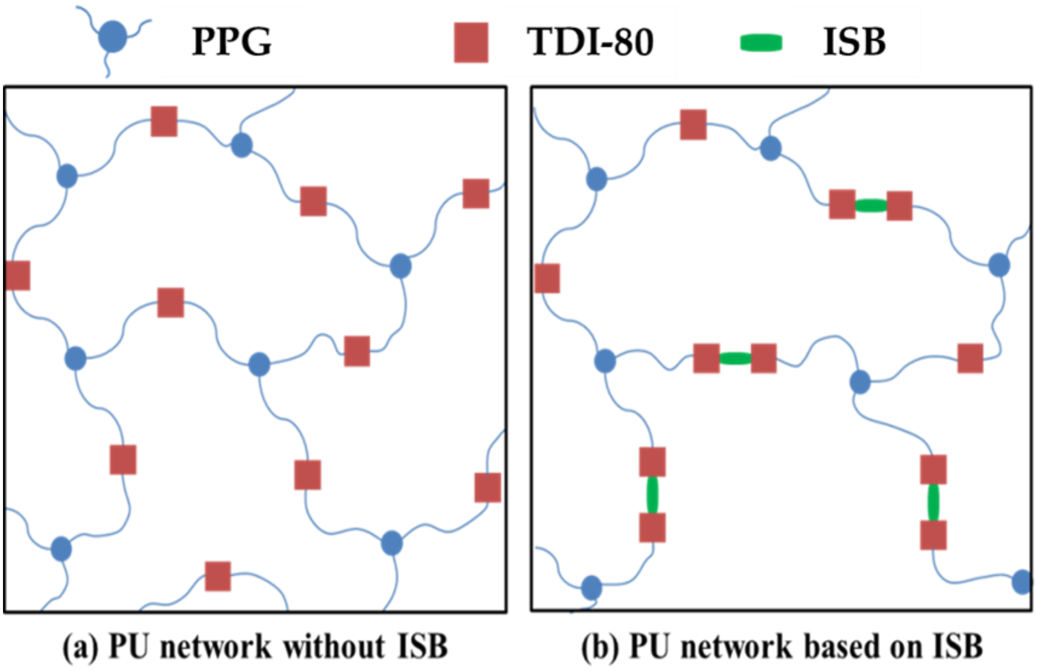

Figure S3. Schematic illustration of crosslinked network structures of (a) PU without ISB and (b) those with ISB.

Table S4. Average cell size, average thickness of cell walls, and the number of cells per unit area of FPUFs with various ISB content before and after thermal treatment.

Sample code	PUF-I0	PUF-I1	PUF-I2	PUF-I3	PUF-I4	PUF-I5
**Before the thermal treatment**						
Average cell size (μm)	449 ± 131	423 ± 134	378 ± 99	416 ± 135	427 ± 131	407 ± 132
Average thickness of cell walls (μm)	34.6 ± 4.5	33.3 ± 5.6	32.1 ± 6.9	31.1 ± 7.4	30.8 ± 8.8	29.6 ± 8.4
Number of cells (cm^{-2})	621	668	727	691	668	703
**After the thermal treatment**						
Average cell size (μm)	412 ± 100	392 ± 87	379 ± 113	389 ± 84	422 ± 142	378 ± 93
Average thickness of cell walls (μm)	34.0 ± 6.3	33.2 ± 6.8	32.5 ± 7.0	32.4 ± 6.3	31.1 ± 4.0	30.1 ± 6.6
Number of cells (cm^{-2})	703	714	738	703	656	785

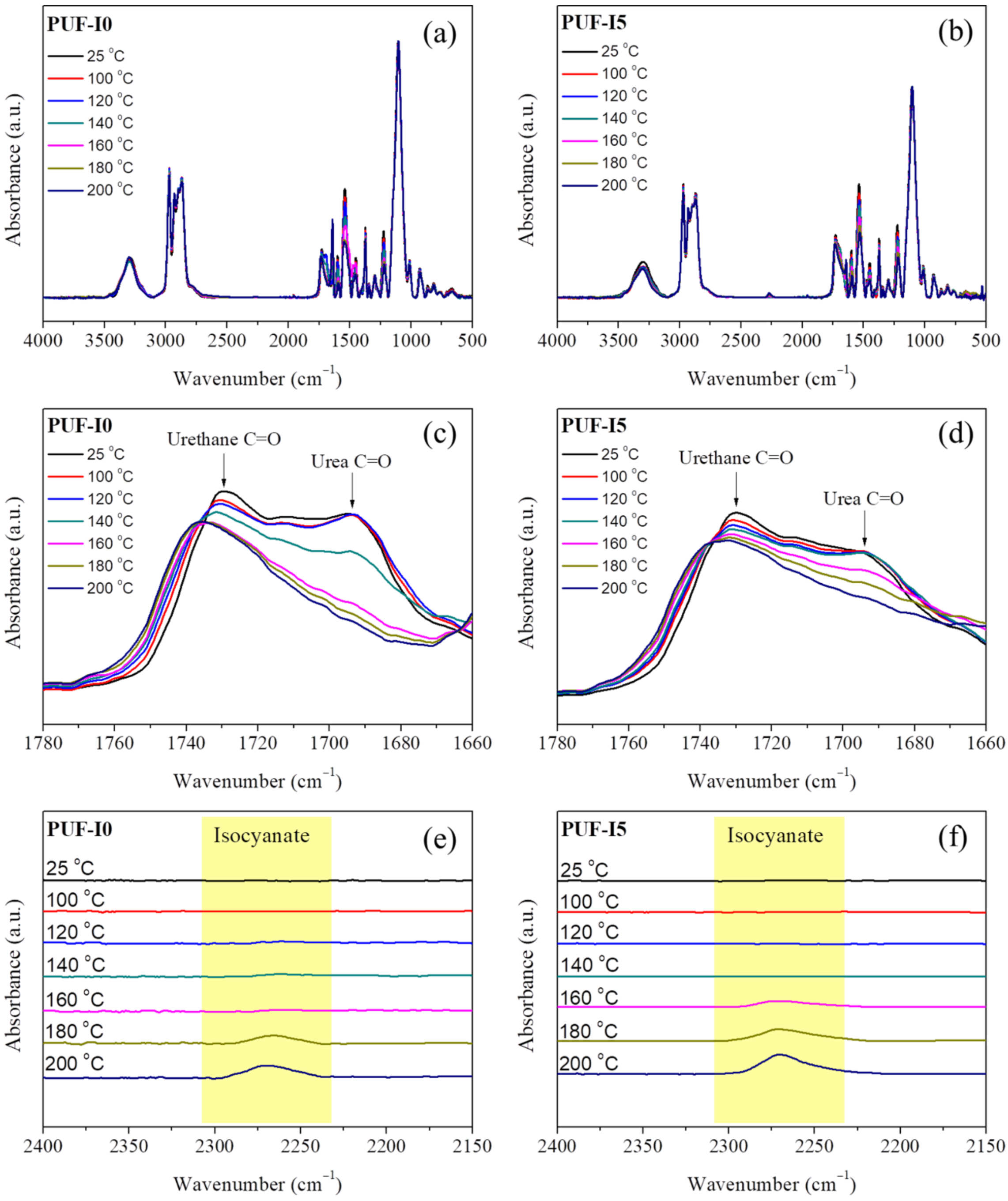

Figure S4. Temperature-dependent FTIR spectra of PUF-I0 and PUF-I5: (a) PUF-I0; (b) PUF-I5; (c) PUF-I0 expanded at 1660 – 1850 cm^{-1}; (d) PUF-I5 expanded at 1660 – 1850 cm^{-1}; (e) PUF-I0 expanded at 2150 – 2400 cm^{-1}; (f) PUF-I5 expanded at 2150 – 2400 cm^{-1}.

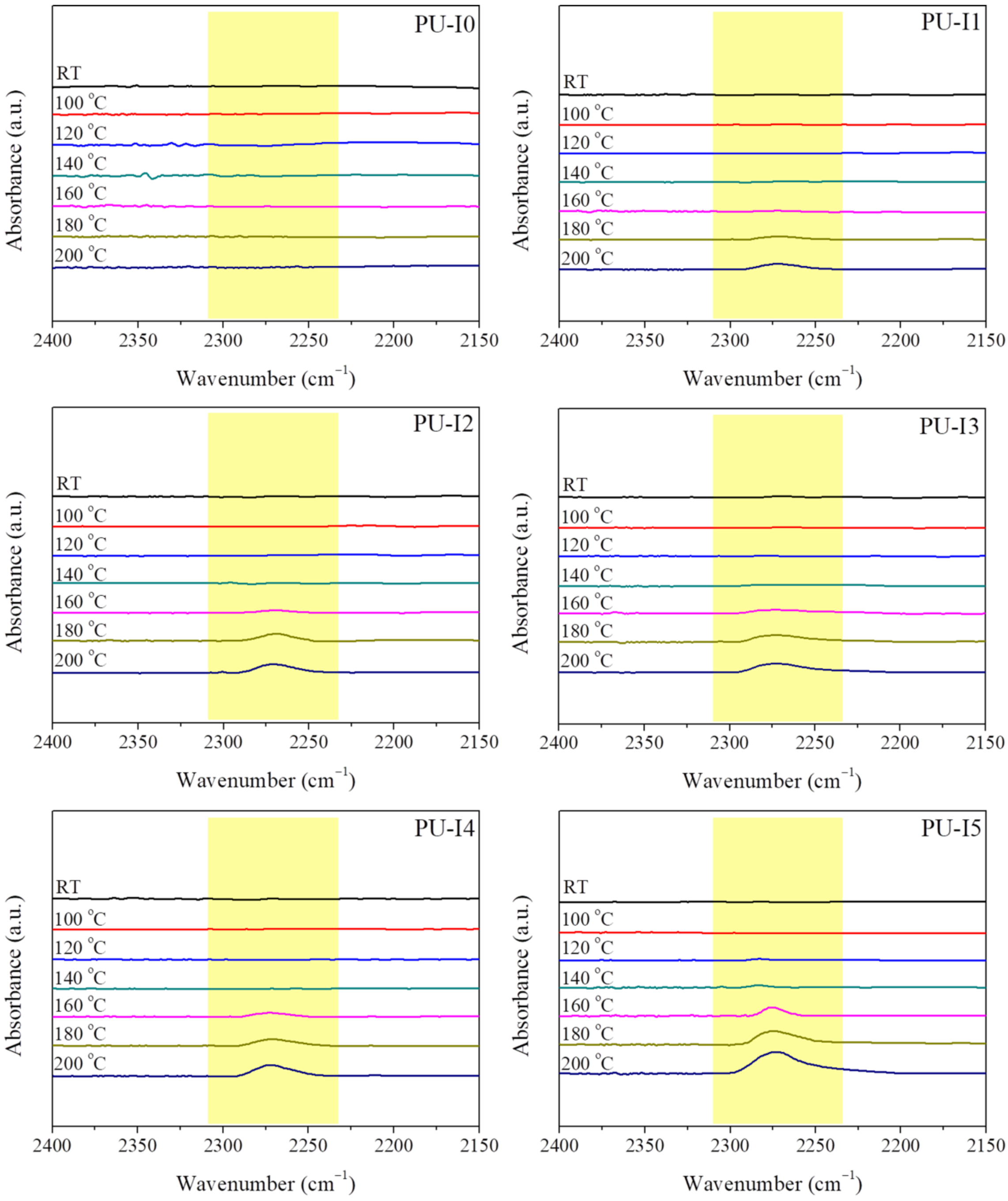

Figure S5. Temperature-dependent FTIR spectra of PU films prepared with different concentrations of ISB.

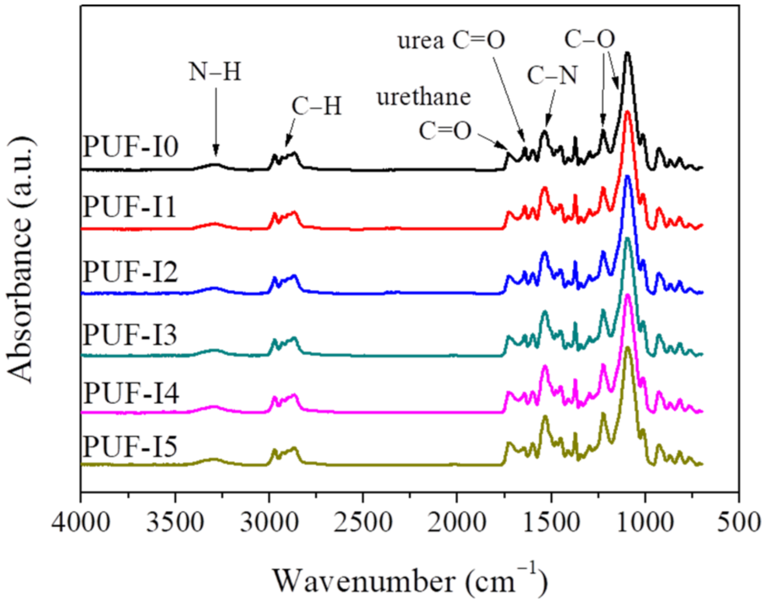

Figure S6. FTIR spectra of different FPUFs after curing at room temperature for 1 h.

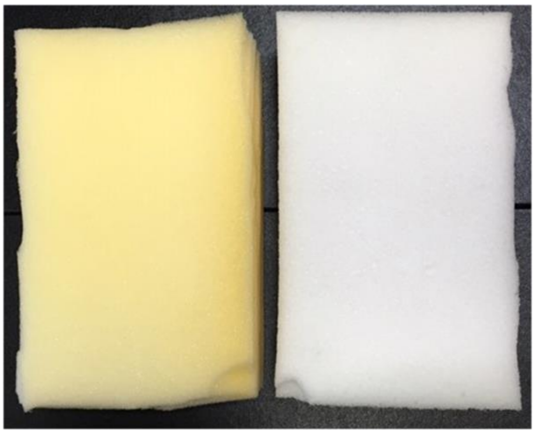

Figure S7. Photographs of PUF-I0 (left) and PUF-I5 (right) left at room temperature for 30 days.

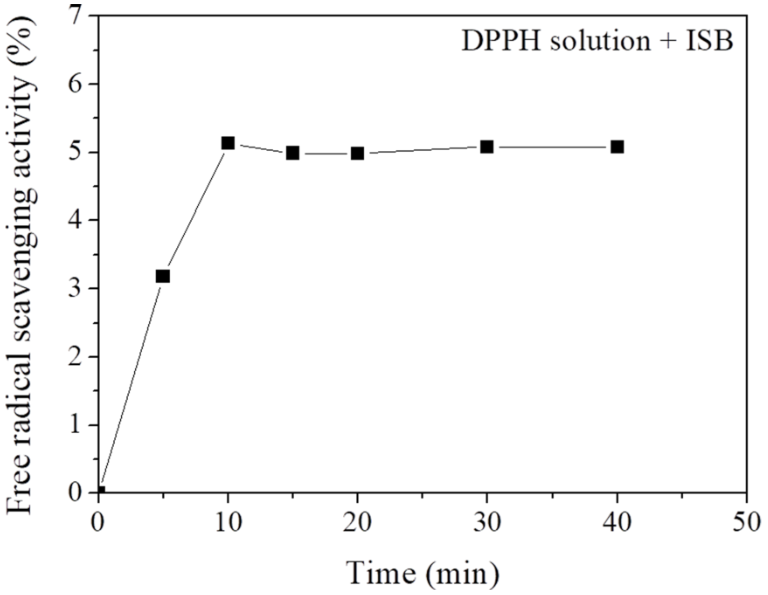

Figure S8. Free radical scavenging activity of ISB solution in methanol (0.3 mM) by DPPH method at room temperature.
